# Single-step immunoblot tests with recombinant protein antigens for detecting IgG and IgM antibodies in Lyme disease

**DOI:** 10.1128/spectrum.00199-26

**Published:** 2026-07-15

**Authors:** Jyotsna S. Shah, Song Liu, Catherine Calalo-Ramos, Mumu Myint, Olivia Lee, Iris Cruz, Elizabeth J. Horn, Ranjan Ramasamy

**Affiliations:** 1 ID-FISH Technology, Milpitas, California, USA; 2 IGeneX 688327 , Milpitas, California, USA; 3 Lyme Disease Biobank, Portland, Oregon, USA; Creighton University, Omaha, Nebraska, USA

**Keywords:** *Borrelia burgdorferi*, immunoblots, immunodiagnostics, Lyme disease Borreliae, serodiagnosis of Lyme disease

## Abstract

Serodiagnosis is important in Lyme disease (LD). Until recently, this commonly required two separate tests termed the standard two-tier tests (STTTs). One-step immunoblot (IB) tests utilizing recombinant protein targets from multiple *Borrelia* species and strains that may simplify the serodiagnosis of LD are now described. Specificity of the IB tests was established with 425 sera from healthy persons and 387 sera from persons with potentially confounding infections and medical conditions. Test sensitivities were determined with (i) 90 sera from patients with different stages of LD from the US Centers for Disease Control and Prevention (CDC), and (ii) 290 prospectively banked sera from patients with acute-stage LD from the Bay Area Lyme Foundation’s Lyme Disease Biobank (BALDB). The IgG IB test was more sensitive than a US Food and Drug Administration (FDA)-cleared STTT for detecting IgG antibodies in stage 1 LD sera from the CDC’s test panel (*P* = 0.002). The diagnostic performance of the IgG and IgM IB tests demonstrated significantly greater overall sensitivity than the comparable FDA-cleared STTTs for detecting IgG and/or IgM antibodies in the 290 BALDB sera (*P* = 0.00004). The IgG IB test was also more sensitive than the IgG STTT for detecting IgG antibodies in the 290 BALDB sera (*P* < 0.000001). The IgG and IgM IB tests were additionally more sensitive than IgG and IgM STTTs for detecting IgG and/or IgM antibodies in BALDB sera from 189 patients with erythema migrans (*P* = 0.03). The IB tests described here form the basis for one-step IgG and IgM IB tests, termed iDart IgG and IgM IB tests, cleared by the FDA for LD diagnosis.

**IMPORTANCE:**

The immunoblot (IB) tests described here are one-step tests that separately detect IgG and IgM antibodies for the serological diagnosis of Lyme disease in the United States. The IB tests are more sensitive than a common standard two-tier test for detecting antibodies in early-stage Lyme disease. This can assist in the diagnosis of Lyme disease when antibodies have not yet reached their maximum concentration in patient sera.

## INTRODUCTION

Lyme disease (LD) is a tick vector-borne disease caused by several species of spirochete bacteria of the genus *Borrelia* termed Lyme disease Borreliae (LDB). The best studied LDB species causing human LD are *Borrelia burgdorferi sensu stricto* (*Bbss*) in the United States and *B. garinii*, *B. afzellii,* and *Bbss* in Europe (1). However, other LDB species have also been reported to cause LD in the United States and Europe (2, 3). The principal US tick vectors (*Ixodes scapularis* in the East Coast and Mid-West, and *I. pacificus* in the West Coast) acquire LDB in a blood meal from wild mammals such as deer and mice and which, after further development in the tick gut, are transmitted to humans during a subsequent blood feed (1, 4, 5). Apart from the detection of a slowly expanding, annular, erythematous skin lesion termed erythema migrans (EM) at the site of a tick bite in LD-endemic areas, the diagnosis of LD principally relies on detecting serum antibodies to LDB antigens (1, 5–7).

In 1995, a standard two-tier test (STTT) procedure for detecting serum antibodies in LD was first established by the US Centers for Disease Control and Prevention (CDC) (6). The first tier of the STTT required an enzyme immunoassay (EIA) with *B. burgdorferi* whole cell lysates or an immunofluorescence assay with *B. burgdorferi* cells (6). A positive or equivocal result on the first-tier test then required a positive result in a more specific second-tier western blot (WB) test for detecting IgM or IgG antibodies against a panel of *B. burgdorferi* antigens for confirmation (1, 6). A WB test for serum IgM antibodies is considered valid only up to 30 days after infection, and scored as positive if antibodies against at least two of the three antigens with relative molecular masses of 23 kDa (outer surface protein C or OspC), 39 kDa (*Borrelia* membrane protein A or BmpA), and 41 kDa (flagellin B or FlaB) are detected (1, 6). The WB test for serum IgG antibodies is considered positive if at least 5 of the 10 antigens of relative molecular masses 18, 23 (OspC), 28, 30, 39 (BmpA), 41 (FlaB), 45, 58, 66, and 93 kDa are recognized (1, 6). STTTs were more recently supplemented with a variety of modified two-tier tests (MTTTs) in the US (8). The MTTTs generally depend on positive reactions in both of the two EIAs that utilize different *B. burgdorferi* target antigens (1, 5, 8). Two-tier serological tests for LD in the United States typically only use target antigens derived from the *Bbss* strain B31, which was originally isolated from a tick (5). Potential advantages as well as difficulties in developing single-step serological tests to replace STTTs and MTTTs in LD have been previously discussed (5, 7, 9).

In a previous pilot study, we investigated the use of recombinant proteins from multiple US and European LDB species, and two different US *Bbss* strains, B31 and 297, in line immunoblots (IBs) as an alternative to WBs with whole *Bbss* cell lysates in the second-tier of a STTT (9). We now compare the diagnostic performance of one-step line IgG and IgM IB tests with FDA-cleared IgG and IgM STTTs for detecting IgG and IgM antibodies in well-characterized serum samples.

## MATERIALS AND METHODS

### Background to the IBs

Gene sequences encoding the recombinant proteins used as target antigens in the IBs were obtained from NCBI GenBank. Recombinant proteins were produced in *Escherichia coli* and purified by affinity chromatography as previously described (9). The nitrocellulose IB strips, with different antigens and control proteins immobilized as discrete lines (that give rise to what are termed bands after development of the IBs), were also prepared and used as reported (9). Two control proteins, C1 which was human immunoglobulin for confirming the addition of alkaline phosphatase conjugated goat anti-human IgG and IgM antibodies, and C2 which was Protein L for confirming the addition of human sera, during the tests were also immobilized as lines on strips (9). The Protein L in C2 additionally served as an internal calibration control so that, after development of the IBs, only bands with a visual intensity equal to or greater than the C2 control were considered positive. The amount of protein L immobilized in IBs was selected to yield a readily readable band compared to the weakest positive IB band obtained with known positive human sera from a CDC research panel of 42 LD sera (9). A pool of sera from patients with confirmed LD served as a positive control, and sera from uninfected persons served as a negative control in every IB test run.

The IgG and IgM IBs both incorporated a proprietary target antigen termed Lyme screen antigen (LSA) composed of the immunodominant regions of the variable surface protein VlsE (10) derived from multiple US and European LDB species.

### The IgG IB test

In addition to LSA, the IgG IBs utilized the following other target proteins (P) of relative molecular mass in kDa for scoring: P23 (OspC), P31 (OspA), P34 (OspB), P39 (BmpA), P41 (FlaB), and P93, all of which had been used in our pilot study (9). Of these, P23 (OspC), P39 (BmpA), P41 (FlaB), and P93 are scoring target antigens used in CDC-recommended IgG STTTs (1, 6). Recombinant proteins derived from seven different US and European LDB species (proprietary) and the two US *Bbss* strains B31 and 297 were immobilized as discrete lines on nitrocellulose membrane strips to yield a group of nine lines of antigens, respectively, in each case for P23 (OspC) and P31 (OspA). A proprietary mix of respective antigens from different US and European LDB species was applied as two separate lines in the case of both P39 (BmpA) and P41 (FlaB). LSA, P34 (OspB), and P93 were applied as single lines.

The IgG IB test was scored as positive if the test serum contained antibodies reacting with LSA and at least two of the following six target antigens: P23 (OspC), P31 (OspA), P34 (OspB), P39 (BmpA), P41 (FlaB), and P93, with band intensities greater than or equal to that of the C2 internal control band. In the P23 (OspC), P31 (OspA), P39 (BmpA), and P41 (FlaB) groups that contained multiple lines of antigens from different LDB species and *Bbss* strains, antibody reaction with one or more lines within each group was scored as a positive reaction for that particular antigen group.

The IgG IB test strip additionally incorporated six antigens: P18, P28, P30, P45, P58, and P66 derived from US *Bbss* strain B31 that are CDC-recommended antigens used for scoring IgG STTTs (1, 6). However, these six antigens were included only for comparative purposes and not used for scoring reactivity in the IgG IBs described here.

### The IgM IB test

The IgM IBs incorporated LSA and P34 (OspB) as single antigen lines; P23 (OspC) and P31 (OspA), each with nine lines of antigens from seven different LDB species and the two *Bbss* strains B31 and 297; and a proprietary mix of P39 (BmpA) and P41 (FlaB) containing antigens from multiple US and European LDB species applied as two lines in both cases. The IgM IB also contained nitrocellulose membrane-immobilized (i) P93 antigen and (ii) protein C10 derived from the C terminus of Osp C (11). P93 and C10 were only included for comparative purposes and not used for scoring the IgM IBs described here.

The IgM IB test was scored as positive if antibodies to LSA and at least two of the five following target antigens were present in the tested serum: P23 (OspC), P31 (OspA), P34 (OspB), P39 (BmpA), and P41 (FlaB). Antibodies reacting with one or more lines in each of the P23 kDa (OspC), P31 (OspA), P39 (BmpA), and P41 (FlaB) groups were taken as a positive reaction for that particular antigen group.

### Specificities of the IgG and IgM IB tests for detecting IgG and IgM

The specificities of the IgG and IgM IB tests for detecting only IgG and IgM antibodies were tested by preincubating the secondary alkaline phosphatase-conjugated goat anti-human IgG and anti-human IgM antibodies (KPL, Gaithersburg, MD, USA) at their usage dilution of 1:10,000 for the anti-human IgG and 1:3,000 for the anti-human IgM with purified human IgG and IgM (Sigma, St. Louis, MO, USA), respectively, at 1 µg/mL. The preincubated secondary antibodies were then tested in IBs with a set of four positive and six negative human sera that had previously given positive and negative results, respectively, in the IgG and IgM IB tests.

### Standard two-tier test for comparison

This study utilized (i) Zeus Scientific’s *Borrelia* VlsE1/pepC10 IgG/IgM EIA as an FDA-cleared first-tier test (12), and (ii) Viramed’s *Borrelia* B31 IgG and IgM ViraStripe tests as an FDA-cleared second-tier test (13).

### Serum samples from patients with Lyme disease, healthy controls, and potentially confounding infections and conditions

Details of the sera used in the study and their sources are summarized in Table 1.

**TABLE 1:** Details of patient and control sera used in the study^*a*^

Source, collection location, and time of collection or receipt as relevant	Sample type	Total number of serum samples
Lyme Disease Biobank of the Bay Area Lyme Foundation, CA (14). Sera collected at East Hampton, NY (2014–2018); Martha’s Vineyard, MA (2015–2016); and Wisconsin Area, WI (2016–2018)	Prospectively banked first sample sera from 290 patients, of whom 142 had EM >5 cm, 47 had EM ≤5 cm diameter, and 101 with physician-assessed LD but no EM.	290
	Sera from healthy controls collected in the same LD-endemic locations.	251
Premarketing test panel sera from the CDC, GA collected in 2008–2012 (15)	Retrospectively banked sera from LD positive US patients, of which 30 were from early acute LD (stage 1) patients with a characteristic EM lesion; 30 from similar stage 1 LD patients in convalescent phase; 10 from early disseminated LD patients with carditis and neuroborreliosis (stage 2); 20 from late-stage LD with arthritis (stage 3).	90
	Sera from healthy controls with 50 from LD endemic areas and 50 from LD non-endemic areas.	100
	Sera from patients with other diseases. Fifteen sera each from patients with fibromyalgia, rheumatoid arthritis, multiple sclerosis, mononucleosis, syphilis, and periodontitis as previously described (15).	90
Additional control US sera from the CDC, GA collected in 2008–2012	Additional sera from healthy controls with 12 from LD endemic areas and 12 from LD non-endemic areas.	24
CDC, GA 2023	Sera from patients with leptospirosis	10
IGeneX Inc, CA collected in 2021–2023	Sera from healthy persons living in CA who voluntarily donated blood.	50
	Sera from US patients with tick-borne diseases other than LD. These were leftover patient samples tested at IGeneX at different times and found to be seronegative for LD by STTTs but seropositive for the following infections: 28 for babesiosis, 48 with bartonellosis, 5 for ehrlichiosis, 7 for anaplasmosis, 22 for rickettsiosis, and 14 for tick-borne relapsing fever.	124
New York Biologics, Southampton, NY received in 2006	Sera from patients with potentially confounding infectious diseases including 12 from patients with human immunodeficiency virus infection, 23 with syphilis, 8 with herpes simplex virus-1 infection, 2 with herpes simplex virus-2 infection, 13 with cytomegalovirus infection, and 27 with Epstein-Barr virus infection.	85
Kamineni Life Sciences, Hyderabad, India received in 2023	Sera from persons with potentially confounding conditions and infections composed of 12 sera from pregnant women and 10 sera from patients with *Helicobacter pylori* infections.	22
Warde Medical Laboratory, Ann Arbor, MI received in ^a^2024 and ^b^2026	Sera from persons with potentially confounding infections. Ten sera each from patients with parvovirus-19 and *Varicella zoster* infections^a^ and 11 sera from patients with toxoplasmosis.^b^	31
BEI Resources, Manassas, VA received in 2020	Sera from persons with potentially confounding viral infections that included four sera from patients with respiratory syncytial virus and 21 sera from patients with influenza virus infections.	25

^*a*^ Superscripts a and b in column 2 indicate the corresponding years of receipt provided in column 1.

### Testing of premarketing panel sera from the CDC

The CDC supplied a set of 280 blinded serum samples, which included sera from patients with LD, patients with other potentially confounding diseases, and healthy controls from both endemic and non-endemic areas (Table 1 and reference 15). IgM and IgG IB tests were performed on all samples, and the results were submitted to the CDC, which subsequently decoded the samples and provided the corresponding first-tier test results obtained using the Zeus *Borrelia* VlsE1/pepC10 IgG/IgM EIA. Virastripe *Borrelia* B31 IgG and IgM tests were then performed on all samples that were positive or equivocal in the first-tier EIA. This enabled compilation and evaluation of the overall STTT results for the CDC serum panel.

### Testing of prospectively banked acute-phase sera from the Bay Area Lyme Foundation’s Lyme Disease Biobank (BALDB)

IgG and IgM IBs, as well as the IgG and IgM STTTs, were performed on 290 acute-phase sera from patients with likely LD, and 251 sera from healthy controls (Table 1 and reference 14). This was done in a blinded manner without prior knowledge of diagnostic findings available at BALDB, and unblinded only after conclusion of tests.

### Resolution of discrepant findings on BALDB acute-phase first collection sera that were positive by IgG and IgM IBs but negative by STTTs

Discrepant analysis was used to resolve the status of the acute-phase sera that were positive in the IgG and/or IgM IBs but negative in the IgG and IgM STTTs. These were further analyzed retrospectively together with data subsequently provided by the BALDB and deemed to be truly positive if (i) the patients donating sera were subsequently revealed to have had an EM lesion >5 cm in diameter, and/or (ii) they had antibodies detectable by any two of the following three different EIA tests, as is done in many MTTTs: (i) Zeus EIA Borrelia VlsE 1/pepC10 IgG/IgM (12), which was used as the first-tier test in the STTTs (where the second tier was an IB test), (ii) the C6 EIA (5, 14), and (iii) the MarDX whole cell EIA for IgG/IgM (9, 14, 16).

### Statistical analysis

Clinical agreement between IgG and IgM IB tests and the STTT results were determined with an online calculator (17). Sensitivities and specificities, as well as two-tailed Pearson chi-square tests (for comparing proportions) and two-tailed McNemar’s exact tests (for paired comparisons of BALDB serum samples) were also calculated online (18). Yate’s correction was applied to chi-square tests when the number in any category for comparison was ≤5.

## RESULTS

### Representative IgG and IgM IBs

**Fig 1:**
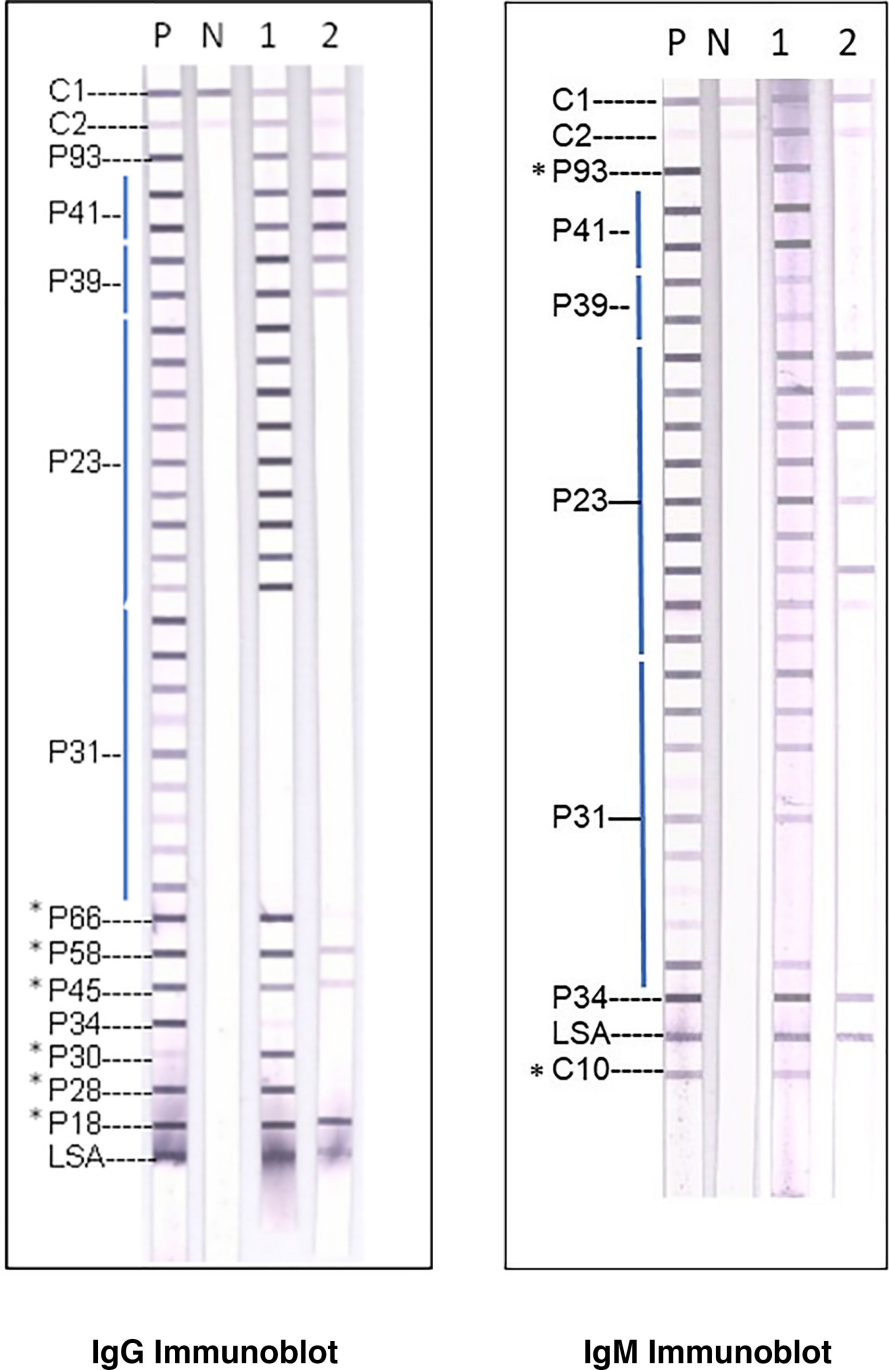
Representative IgG and IgM IBs. P—positive control, pool of positive sera from patients with LD; N—negative control, pool of sera from healthy persons; 1—strongly positive test serum from a patient with LD; 2—weakly positive test serum from a patient with LD; Control proteins C1 and C2, and the scoring LDB antigens are explained in Methods. Asterisks show LDB antigens used in STTTs that were also present in the IBs but not used for scoring, as explained in Materials and Methods.

IgG and IgM IBs with representative serum samples from LD patients are shown in Fig. 1. The IBs provided evidence to suggest that antibodies from different patients differentially recognize homologous target antigens derived from varying LDB species and strains. For example, the weakly positive patient serum 2 shown in Fig. 1 had IgM antibodies that respectively recognized only some of the nine P23 (OspC) proteins, while the strongly positive patient serum 1 only recognized some of the nine P31 (OspA) proteins in the IgM IB.

### Specificity of the secondary goat anti-human immunoglobulin antibodies

The reactivities of the goat anti-human IgG and goat anti-human IgM antibodies used in the IB tests with known positive LD patient sera were only blocked with human IgG and human IgM, respectively, and not vice versa. This confirmed the required specificity of recognition of only IgG antibodies in the IgG IB test and only IgM antibodies in the IgM IB test.

### Analytical specificity

None of the 387 sera from persons with health conditions and unrelated diseases (Table 1) that were potentially expected to have antibodies that cross-react with LDB antigens (5) were found to be positive in the IgG IB test. The 387 sera included 124 from patients with other US tick-borne diseases, and additionally sera from patients with viral and bacterial infections, as well as conditions like rheumatoid arthritis, fibromyalgia, and pregnancy (Table 1). In the 387 sera, only one parvovirus-19 positive patient’s serum was positive in the IgM IB test, yielding an estimated specificity of 99.7% (with a 95% confidence interval of 98.3–100%) for the IgM IB test, but the same serum was negative in the IgG test.

Tables 2 and 3 summarize IgG and IgM IB test results, respectively, with serum samples collected from a population of 313 healthy individuals living in LD-endemic areas, and 112 samples collected from healthy individuals living in LD-non-endemic areas of the United States. The estimated specificities of the IgG IB test were 99.4% and 100%, respectively, in endemic and non-endemic areas of the United States. The estimated specificity was 100% for the IgM test in both endemic and non-endemic areas of the United States.

**TABLE 2:** IgG and IgM IB test results with 313 sera from healthy individuals living in LD endemic areas

Source of sera	No. of sera tested	No. of sera with IgG antibodies	No. of sera with IgM antibodies
US CDC	62	0	0
BALDB	251	2	0
Total number of sera	313	2	0
Specificity (95% confidence interval)		99.4% (97.4–99.9%)	100% (98.5–100%)

**TABLE 3:** IgG and IgM IB test results with 112 sera from healthy individuals living in LD non-endemic areas

Source of sera	No. of sera tested	No. of sera with IgG antibodies	No. of sera with IgM antibodies
US CDC	62	0	0
IGeneX California	50	0	0
Total number of sera	112	0	0
Specificity (95% confidence interval)		100% (95.9–100%)	100% (95.9–100%)

### Sensitivities of the IgG and IgM IBs tests with 90 US CDC pre-marketing test panel sera obtained from US patients with different stages of LD

The US CDC pre-marketing test panel (15) contained sera collected from patients diagnosed with different stages of LD: early localized infection with an EM lesion (stage 1), early disseminated infection with carditis or neuroborreliosis (stage 2), and late-stage LD with arthritis (stage 3). The 60 stage 1 sera comprised 30 from patients with an EM lesion before treatment and another 30 from patients with an EM lesion but collected 10–35 days following the patients’ initial diagnosis and after commencing antibiotic treatment (15). First tier of the STTTs on the 90 pre-marketing test panel sera had already been performed by the US CDC, but the findings were only made available to the authors after they had independently completed the IB tests. Composite findings from the IgG IB and IgM IB tests and the corresponding IgG and IgM STTTs are summarized in Table 4.

**TABLE 4:** Findings with IgG and IgM IB tests and the corresponding IgG and IgM STTTs in 90 sera of patients with different stages of LD from the US CDC pre-marketing serum panel

	Stage 1		Stage 2		Stage 3		Overall	
No. of sera	60		10		20		90	
Comparison of IgG IB and IgG STTT findings in different LD stages								
**Test**	**IgG IB**	**IgG STTT**	**IgG IB**	**IgG STTT**	**IgG IB**	**IgG STTT**	**IgG IB**	**IgG STTT**
No. of positive	35	18	9	9	20	20	64	47
No. of negative	25	42	1	1	0	0	26	43
Sensitivity (95% confidence interval)	58.3% (44.9–70.1%)	30.0% (19.2–43.4%)	90.0% (54.1–99.5%)	90.0% (54.1–99.5%)	100% (80.0–100%)	100% (80.0–100%)	71.1% (60.4–79.9%)	52.2% (41.5–62.8%)
Comparison of IgM IB and IgM STTT findings in different LD stages								
**Test**	**IgM IB**	**IgM STTT**	**IgM IB**	**IgM STTT**	**IgM IB**	**IgM STTT**	**IgM IB**	**IgM STTT**
No. of positive	38	39	8	9	3	15	49	63
No. of negative	22	21	2	1	17	5	41	27
Sensitivity (95% confidence interval)	63.3% (49.8–75.1%)	65.0% (51.5–76.6%)	80.0% (44.2–96.5%)	90.0% (54.1–99.5%)	15.0% (0.4–38.9%)	75.0% (50.6–90.4%)	54.4% (43.6–64.9%)	70.0% (59.3–79.0%)
IgG and/or IgM IB findings compared with IgG and/or IgM STTTs in different LD stages								
**Test**	**IB**	**STTT**	**IB**	**STTT**	**IB**	**STTT**	**IB**	**STTT**
No. of positive	49	42	9	10	20	20	78	72
No. of negative	11	18	1	0	0	0	12	18
Sensitivity (95% confidence interval)	81.7% (69.1–90.1%)	70.0% (56.6–80.8%)	90.0% (54.1–99.5%)	100% (65.5–100%)	100% (80.0–100%)	100% (80.0–100%)	86.7% (77.5–92.6%)	80.0% (70.0–87.4%)

The IgG IB test was significantly more sensitive than the IgG STTT in stage 1 LD (*χ*^2^ = 9.77, *P* = 0.002), with the tests respectively detecting IgG antibodies in 58.3% versus 30.0% of the US CDC’s stage 1 LD serum test panel. However, the IgM IB test and IgM STTT were not significantly different in their abilities for detecting IgM antibodies in stage 1 LD sera (*χ*^2^ = 0.04, *P* = 0.84). Considering all three stages taken together, the IgM STTT was apparently significantly more sensitive than the IgM IB test (*χ*^2^ = 4.6, *P* = 0.03), largely due to the IgM STTT’s greater sensitivity in detecting stage 3 LD (*χ*^2^ = 14.5, *P* = 0.0001). However, the use of STTTs to detect IgM antibodies in late-stage LD is not recommended due to greater cross-reactivity and the expected conversion to IgG antibodies in late-stage LD (1, 5, 6), thus invalidating a comparison between the IgM IB test and IgM STTT in stage 3 LD and in all three stages combined. While the two IB tests detected a higher proportion of either IgG and/or IgM antibodies in stage 1 LD than the STTT tests (81.7% vs 70.0%), the difference did not quite achieve statistical significance (*χ*^2^ = 2.23, *P* = 0.14), which can be due to the relatively small number (60) of stage 1 LD samples in the US CDC pre-marketing panel. Analysis of 290 early LD serum samples from the BALDB in the same way revealed statistically significant greater sensitivity of the IB tests compared to STTTs (Tables 7 and 8 below).

### Findings with IB tests and the STTTs with prospectively banked BALDB sera from patients with clinical signs and symptoms of LD

The IB tests and STTTs were performed blindly without prior knowledge of any serological test findings previously available at the BALDB (14). Findings with the 290 sera (details of which are provided in Table 1) are summarized in Tables 5 to 7.

**TABLE 5:** Results with the IgG IB test and the IgG STTT on 290 sera from the BALDB^*a*^

Test		IgG STTT	
		Positive	Negative
IgG IB	Positive	19	36
	Negative	1	234
	Column totals	20	270

^*a*^ Positive percent agreement (95% confidence interval) = 95.0 (76.4–99.1). Negative percent agreement (95% confidence interval) = 86.7 (82.1–90.2). Overall percent agreement (95% confidence interval) = 87.2 (82.9–90.6).

**TABLE 6:** Results with the IgM IB test and the IgM STTT on 290 sera from the BALDB^*a*^

Test		IgM STTT	
		Positive	Negative
IgM IB	Positive	35	12
	Negative	8	235
	Column totals	43	247

^*a*^ Positive percent agreement (95% confidence interval) = 81.3 (66.1–91.1). Negative percent agreement (95% confidence interval) = 95.1 (91.5–97.3). Overall percent agreement (95% confidence interval) = 93.1 (89.4–95.6).

**TABLE 7:** Results with the IgG and/or IgM IB test and the IgG and/or IgM STTTs on 290 sera from the BALDB^*a*^

Test		IgG and/or IgM STTTs	
		Positive	Negative
IgG and/or IgM IBs	Positive	47	22
	Negative	2	219
	Column totals	49	241

^*a*^ Positive percent agreement (95% confidence interval) = 95.9 (84.9–99.3). Negative percent agreement (95% confidence interval) = 90.9 (86.3–94.1). Overall percent agreement (95% confidence interval) = 91.7 (87.8–94.5).

The IgG IB test was significantly more sensitive than the STTT for detecting IgG antibodies in the acute phase sera from BALDB (19.0% vs 6.9%; *P* < 0.000001) (Table 5). There was no significant difference between the IgM IB test and the IgM STTT for detecting IgM antibodies (*P* = 0.5) (Table 6). Considering the detection of either IgG and/or IgM antibodies in the BALDB sera, the positivity rate for the IB tests of 23.8% was significantly higher than the positivity rate of 16.9% for STTTs (*P* = 0.00004) (Table 7). The overall percent agreement between IBs and the STTTs for detecting either IgG and/or IgM antibodies in the BALDB acute-phase sera was a high 91.7% (Table 7).

Table 7 shows that 22 sera that were positive in the IB tests for either IgG and/or IgM antibodies were negative for either IgG and/or IgM antibodies by the IgG and IgM STTTs. Discrepancy analysis confirmed that 21 of these 22 sera were truly positive for LD because 17 of the 21 sera were from patients with EM >5 cm diameter and the other four of the 21 sera tested positive in two different EIAs. The positivity rate with IB tests for either IgG and/or IgM antibodies of 23.4% continued to remain significantly higher than the positivity rate for STTTs of 16.9% after the discrepancy analysis (*P* = 0.00007).

The relative sensitivities of the IB tests and STTTs for detecting IgG and IgM antibodies in the acute phase BALDB sera were further compared using data on the presence of EM lesions >5 cm in diameter or ≤5 cm in diameter or physician-assessed LD symptoms but without EM in donors, which were retrospectively made available to the investigators by BALDB (Table 8).

**TABLE 8:** Performance of IgG and/or IgM IB tests and the IgG and/or IgM STTTs with BALDB sera from patients with EM and those with LD symptoms but no detected EM

Patient characteristics	Total no. of sera	No. of sera IgG and/or IgM IB test positive	No. of sera IgG and/or IgM STTT positive	Significance of difference, *χ*^2^ value and probability
EM lesion >5 cm in diameter	142	51 (35.9%)	35 (24.6%)	*χ*^2^ = 4.3; *P* = 0.04
EM lesion ≤5 cm in diameter	47	6 (12.8%)	4 (8.5%)	Yates *χ*^2^ = 0.5; *P* = 0.7
EM lesion (all)	189	57 (30.2%)	39 (20.6%)	*χ*^2^ = 4.5; *P* = 0.03
Symptomatic LD with suspected tick bite but no EM	101	12 (11.9%)	10 (9.9%)	*χ*^2^ = 0.2; *P* = 0.65

The results with sera from the BALDB show that IgG and IgM IB tests used together were significantly more sensitive than IgG and IgM STTTs used together for detecting antibodies in patients with well-defined EM lesions, that is, in patients with confirmed early-stage LD (Table 8). They are also consistent with findings that the IgG tests performed better than IgG STTTs with acute-phase sera from BALDB and LD stage 1 sera from the CDC pre-marketing test panel as shown in Tables 4 and 5.

## DISCUSSION

The high specificities of the IgG and IgM IB tests with (i) sera of patients with other common tick-borne bacterial and parasitic diseases that often share common non-specific symptoms with LD, (ii) sera from persons with other known potentially confounding infections and medical conditions (5), as well as (iii) sera from healthy persons living in LD-endemic and non-endemic areas, are broadly equivalent to the specificities of STTTs (5), demonstrating the utility of IB tests for the laboratory diagnosis of LD. The two IB tests used together had a higher sensitivity in early LD than the comparative STTT and significantly so in particular for detecting IgG antibodies. The findings here also show that the IB tests did much better in early-stage LD with EM than the comparative STTT. Serodiagnosis in early stages of LD is a recognized shortcoming (5, 19, 20). In spite of their high sensitivity in early-stage LD, the IgG and IgM IB tests in combination were estimated to detect antibodies in only 35.9% of patients with acute early-stage LD with EM lesions >5 cm in diameter. Therefore, investigating further lowering of the threshold for detecting antibodies in early LD can be useful.

The STTTs differed from IBs in not using P31 (OspA) and P34 (OspB) as target antigens (1, 6). The use of P31 (OspA) and P34 (OspB) as target antigens and antigens from different LDB species could have contributed to the high sensitivities of the IB tests in early LD. Findings with representative patient sera shown here, and early independent observations (21, 22), are consistent with the importance of using target antigens from different LDB species and strains for the serodiagnosis of LD in the United States. Cross-reactivity data in IB tests with rabbit antisera raised separately against *Bbss* strains B31 and 297, *B. afzelii*, *B. californiensis*, *B. garinii*, *B. spielmanii*, and *B. valaisiana* also support this assertion (9). These observations suggest that additional investigations on the prevalence of infections with LDB species other than *Bbss* may be helpful in better understanding the epidemiology of LD in the United States.

Both IgG and IgM IB tests require a single experimental step compared with the two distinct steps needed for FDA-cleared STTTs and MTTTs. The detection of antibodies to LSA and the other LDB antigens in the IgG and IgM IB tests can be considered to be respectively equivalent to the first and second tier tests of STTTs. The one-step IB tests may therefore simplify the serological diagnosis of LD. They are also readily adaptable for machine reading for greater objectivity and automation.

The IgG and IgM IB tests described here form the bases for US FDA-cleared iDart IgG and IgM IB tests (510k numbers K233367 and K242872, respectively) for detecting IgG and IgM antibodies in patient sera for the laboratory diagnosis of LD. All the information needed for performing the IgG and IgM IB tests will be made readily available with the iDart IgG and IgM test kits to all users.

## Supplementary Material

Reviewer comments

## SUPPLEMENTAL MATERIAL

OPEN PEER REVIEW reviewer-comments.pdf An accounting of the reviewer comments and feedback.

ASM does not own the copyrights to Supplemental Material that may be linked to, or accessed through, an article. The authors have granted ASM a non-exclusive, world-wide license to publish the Supplemental Material files. Please contact the corresponding author directly for reuse.
