# Supplementary material for: Single-step immunoblot tests with recombinant protein antigens for detecting IgG and IgM antibodies in Lyme disease
Source: Microbiol Spectr. 2026 Jul 15;14(8):e00199-26. doi: 10.1128/spectrum.00199-26 (PMC13436020; doi:10.1128/spectrum.00199-26)
Supplement: Supplementary file 1 [file reviewer-comments.pdf]

AMERICAN SOCIETY FOR MICROBIOLOGY | Microbiology Spectrum

Single Step Immunoblot Tests with Recombinant Protein Antigens for Detecting IgG and IgM Antibodies in Lyme Disease

Jyotsna Shah, Song Liu, Catherine Calalo-Ramos, Mu Myint, Olivia Lee, Iris Cruz, Elizabeth Horn, and Ranjan Ramasamy

Corresponding Author(s): Ranjan Ramasamy, ID-FISH Technology Inc

Review Timeline:

Submission Date:	March 13, 2026
Editorial Decision:	March 31, 2026
Revision Received:	April 11, 2026
Accepted:	April 20, 2026

Editor: Travis Bourret

Reviewer(s): The reviewers have opted to remain anonymous.

Transaction Report:

(Note: With the exception of the correction of typographical or spelling errors that could be a source of ambiguity, letters and reports are not edited. The original formatting of letters and referee reports may not be reflected in this compilation.)

DOI: https://doi.org/10.1128/spectrum.00199-26

Re: Spectrum00199-26 (Single Step Immunoblot Tests with Recombinant Protein Antigens for Detecting IgG and IgM Antibodies in Lyme Disease)

Dear Dr. Ranjan Ramasamy:

Thank you for the privilege of reviewing your work. Below you will find my comments, instructions from the Spectrum editorial office, and the reviewer comments.

I am pleased to inform you that your manuscript has been editorially accepted for publication. However, there are a few additional questions in the submission form that need to be answered before the final decision. Once these are completed, please return your submission so that I can move your paper forward to acceptance.

Data availability: ASM policy requires that data be available to the public upon online posting of the article, so please verify all links to sequence records, if present, and make sure that each number retrieves the full record of the data. If a new accession number is not linked or a link is broken, provide Spectrum production staff with the correct URL for the record. If the accession numbers for new data are not publicly accessible before the expected online posting of the article, publication may be delayed; please contact production staff (Spectrum@asmusa.org) immediately with the expected release date.

Publication Fees: For information on publication fees and which article types are subject to charges, visit our website. If your manuscript is accepted for publication and any fees apply, you will be contacted separately about payment during the production process; please follow the instructions in that e-mail. Arrangements for payment must be made before your article is published.

ASM Membership: Corresponding authors may join or renew ASM membership to obtain discounts on publication fees. Need to upgrade your membership level? Please contact Customer Service at Service@asmusa.org.

The ASM Journals program strives for constant improvement in our submission and publication process. Please tell us how we can improve your experience by taking this quick Author Survey.

Thank you for submitting your paper to Spectrum.

Sincerely,
Travis Bourret
Editor
Microbiology Spectrum

RESPONSE TO THE REVIEWERS' COMMENTS

Reviewer #1 (Major Comments for the Author):

1.1 This study shares performance data on the IDart IgG and IgM immunoblot tests, which have recently been cleared for marketing by FDA. The study is extensive, utilizes multiple specimen sources with qualified specimens spanning the spectrum of Lyme Disease stages. The technical approach used appears similar to that of other blotting assays but includes additional recombinant antigens that are proposed to improve sensitivity early in infection while retaining equal specificity as seen with conventional STTT. This is a very important point given the well-known limitations with lab diagnoses early in an infection. The improved sensitivity performance appears to be significant, however there still remain considerable negative results from infected patients, demonstrating that the improvement is incremental and will still miss active early cases.

We appreciate these comments. In the revised manuscript, we have improved the comparison of the IBs with STTTs throughout. We also highlight the need for trying to further improve detection sensitivity in early LD when antibodies have not reached maximum concentration in L382-388.

1.2 The claims are made that the tests can 'reduce the time, effort and cost involved in the laboratory diagnosis of LD', though there are no data presented that address time, effort and cost. The assays appear to require manual performance and interpretation (a reader appears to be available), similar as with STTT blots; a cost savings can be generated by not needing the initial screening EIA to determine whether blots are required. Given most Lyme screening tests are negative though, this approach may result in many more immunoblots being performed than with traditional STTT. Also, given that many labs are switching to MTTT algorithms where automation is a key factor, the comments about reduced time, effort and cost may need additional commentary. Comparison with MTTT performance on the specimen collection would have been very helpful given the widespread adoption occurring with MTTT approaches.

We understand these comments and have suitably modified the Discussion L400-405. We were unable to do comparisons with MTTTs here but plan to do this in the future.

1.3. The manuscript is generally well-written and provides important information, especially on the value of the additional antigens being tested that may contribute to improved performance.

We appreciate the comment

Reviewer #1 (Minor Comments for Author (Required):

1.4 Table 1: there is a discrepancy in the number of specimens included in Lyme Disease BioBank under a): the total of specimens listed equals 288 (140+47+101), not 290. Please update.
Table 1 could be expanded somewhat to include all categories of specimens tested, by adding the 376 sera as another line, so all specimens tested are framed in one table.
Line 186-203: It would have been of interest in having sera included that are positive for toxoplasmosis, given their very common occurrence.

Thank you for this. We have corrected the specific error in Table 1. Table 1 has also been expanded as helpfully suggested to include the other serum controls that were previously mentioned in the text. We have added findings with 11 sera of patients with toxoplasmosis that we obtained from the Warde Medical Laboratory (added to Table 1). All 11 sera were negative in the IgG and IgM IB tests.

1.5 Also in this section mention should be made that all of the specimens positive for other tick-borne diseases were tested for Lyme and found to be negative, this is understood but to the reader the question arises.

We appreciate this comment and have stated in Table 1 where sera from other tick-borne diseases are described that these would have been tested by STTTs for LD and found to be negative. The section on Analytical Specificity under Results (L263-271) mentions more clearly that the 376 sera from persons with other tick-borne diseases, other potentially confounding infections and pertinent medical conditions when tested in the iDart IgG IB tests were found to be negative. One parvovirus infection serum was however positive in the iDart IgM IB test.

1.6 Table 5: The legend mentions that 290 specimens were included but in 5a the total is 270, 5b, 247 and 5c, 241. Perhaps clarify the differences.

Our previous presentation in Tables 5a, 5b and 5c was not sufficiently clear and led to this misreading. We have now clarified in the revised manuscript that 270, 247 and 241 were the totals for the negative columns and the total number of specimens tested in all three Tables was 290 as stated in the Table titles i.e. the sum of all positive and negative specimens.

Reviewer #3 (Major Comments for the Author):

In the manuscript "Single step immunoblot tests with recombinant protein antigens for

detecting IgG and IgM antibodies in Lyme disease" (JCM01667-25), Shah et al. provide evaluation data for a recently FDA-cleared immunoblot assay for detection of Borrelia burgdorferi infection (IgM- and IgG-specific antibodies detected in separate immunoblots). The authors cite this modality as advantageous as it could reduce the requirement of confirmatory (second-tier) assays that have proven necessary due to past issues with specificity in first-tier screening assays. Multiple antigen preparations for four prominent immunodiagnostic B. burgdorferi antigens are included on both the IgG and IgM immunoblots. The authors provide a robust data set that included approximately 536 specimens from individuals with characterized Lyme borreliosis (approximately 186 of these specimens were convalescent draws from a same given patient), 376 sera from individuals with conditions (infection, autoimmune, other) that have been demonstrated to cross-react with Lyme borreliosis serology, and approximately 425 healthy individuals (around 75% of those residing in areas endemic for Lyme borreliosis). The comparator assay(s) in this evaluation was a combination of FDA-cleared assays (with differing borrelial antigen targets) to fulfil current first-tier and second-tier testing criteria. Data reveal equivalent performance of the testing modality, with potential increased IgG sensitivity in early disease.

The manuscript was fairly well written and easy to follow. A couple of adjustments can be made to improve the quality of the document (particularly as they apply to Materials and Methods). Specific comments follow:

We appreciate these comments. In the revision we have deleted reference to the use of 156 convalescent patient sera Bay Area Lyme Foundation Biobank (Table 1 and throughout) to avoid confusion with the acute phase sera. Convalescent phase sera results were also not essential for Discrepancy analysis as 17 of 21 sera classed as True Positives had EM lesions and the other 4 were positive by the two EIA criteria (L348-355).

3.1 With respect to the Abstract, presentation of actual data (rather than a summary of methods) can assist in the quality of this paragraph

We appreciate this comment and have now better summarized the findings in the abstract taking into consideration that the Abstract has to be ≤250 words according to the journal requirement.

3.2 Line 76, recommend "borreliae" without italics;

Thank you for highlighting this and we have corrected it

3.3 Much of the first paragraph of the Introduction section can be omitted or condensed (lines 77-86) without losing message.

We appreciate this comment and have condensed this part of the introduction. We retained a single sentence on US tick vectors to assist non-specialist readers and to provide a more holistic introduction.

3.4 From lines 119-149, this Reviewer was craving more information about the strains/species used and how this assay could potentially translate to use in Europe (the authors do state that it is FDA-cleared in the United States). Are B31 and 297 European strains or United States strains? Lines 132-133, which/how many immunodominant regions of VlsE? Which US and European species (the authors claim multiple species)? Is this proprietary information? If so, please state. If not, a supplementary table may be indicated;

We understand these comments: (i) in L104 of the Introduction and in Methods L 145 we have now further emphasized that *Bbss* B31 and 297 were isolated in the US, (ii) We now explain in more detail the use of homologous target antigens from five different US and European LDB species, the US *Bbss* B31 and 297 strains and the Lyme Screen Antigen (LSA) in L134-151 in the Methods and clearly stating which aspects are proprietary information.

3.5 Lines 126-127, please provide additional protocol details on how proteins were produced for inclusion on immunoblots? Alternatively, was this done using the procedure stated in reference 9? Alternatively, was this proprietary in nature? If so, please state;

We thank the reviewer for these comments. Recombinant proteins were produced in E. coli and purified as previously described in reference 9. This is now better clarified in Methods in L116 to 132 as suggested.

3.6 Lines 142-144, what was the derivation of the criterion for IgG-positive result? Is there rationale for including P93 (rather than several other CDC-recommended IgG bands) within this criterion?

We appreciate this relevant comment. Incorporation of the non-scoring CDC antigens in the IBs was anticipated to help scoring by both the iDart and CDC STTT criteria in one test strip. Lines 152-158 in the revised manuscript now better explain the IgG scoring criteria. P93 was retained for scoring in the IgG IB because specificity and sensitivity analysis had shown its particular additional value.

3.7 Lines 156-158, what was the derivation of the criterion for IgM-positive result?

We appreciate this relevant comment. Lines 170-175 in the revised manuscript now better explain the IgM scoring criteria. The scoring antigens in the IgM IBs were selected through sensitivity and specificity analysis.

3.8 Table 1, should the BALDB erythema migrans >5 cm patient number be 142, rather than 140 (Table 6)?

Thank you for highlighting this error and it has been rectified in Table 1. The title of Table 6 has also been further clarified

3.9 Line 220, as part of the adjudication criteria for investigational/reference discrepancies, the authors list performance on three EIA, with two out of three common results indicating the reference result. The Borrelia VlsE 1/pepC10 IgG/IgM appears to be one of these adjudicators...as well as a component of the reference result (line 173). Please clarify;

Thank you for this comment. The Zeus Borrelia VlsE 1/pepC10 IgG/IgM EIA was used as the first-tier test in the FDA-cleared comparator STTT and Viramed Borrelia B31 IgG and IgM ViraStripe IB tests as the FDA-cleared second-tier test. The discrepancy analysis was done on sera where the STTTs were negative overall. If the first tier Borrelia VlsE 1/pepC10 IgG/IgM EIA was positive and the second tier Viramed Borrelia B31 IgG and IgM ViraStripe tests negative the serum would have been classified as negative in the comparative STTT.

In the Discrepancy analysis where the iDart IB test was positive and the STTT negative we took a positive Zeus Borrelia VlsE 1/pepC10 IgG/IgM EIA test for the discrepant serum in the tier 1 of the STTT as one of the two different EIAs (out of the three EIAs selected as explained better now in the section of Resolution of Discrepancy Findings under Methods) that needed to be positive as in the presently accepted for positivity in MTTTs that depend on EIAs only.

3.10 Do data in Tables 5a, 5b, and 5c reflect any adjudication; and,

Data in Tables 5a, 5b and 5c were not subject to discrepancy analysis (adjudication). Discrepancy analysis process is now further clarified and emphasized in L218-228 and 348-355.

3.11 With respect to lines 372-375, the idea of differential serological profiles for B. burgdorferi dates back around three decades (PMID 8014485).

We appreciate this comment cited this 1994 reference as new reference 21 .

Reviewer #3 (Minor Comments for Author (Required)):

None

Reviewer #4 (Major Comments for the Author):

4.1 Line 50: This test has been "cleared" by FDA. It was not an FDA-approved test.

We appreciate this comment and have used the term ‘cleared’ throughout the revised article.

4.2 What is the rationale for inclusion of such a wide variety of proteins from multiple species of B. burgdorferi when U.S. infections stem largely from B. burgdorferi sensu stricto infection? Likewise, what is the rationale and justification for inclusion of 9 different P23 and P31 proteins in the blots? Given the number of samples tested please present data on the incidence of reactivity to the various bands to support their inclusion? Could the P23 and P31 proteins be combined into two bands (similar to the LSA band)?

Thank you for this comment. Although most LD cases in the US are caused by *B. burgdorferi* sensu stricto (Bbss), several other Lyme disease Borrelia (LDB) species have now been reported within the United States. In addition, U.S. residents frequently acquire LD during travel to Central and South America and Europe, where LDB species other than Bbss predominate. Including proteins from multiple species therefore increases the diagnostic breadth and ensures that the assay can detect infections acquired both domestically and abroad.

Regarding the P23 and P31 antigens, our data indicate that combining these proteins into two consolidated bands results in reduced sensitivity. In contrast, using homologous antigens individually provides better discrimination and stronger reactivity patterns. This differential reactivity is demonstrated in Figure 1, summarized in L243–247 and is consistent with our earlier findings using rabbit antisera raised against various LDB species (citation 9 and L392–399). Additional unpublished results from testing numerous patient sera in our laboratory further support the value of including multiple homologous antigen targets where possible.

A larger study involving well-characterized, genotyped LDB infections would be required to quantify the incidence of reactivity to each individual band more formally. However, such an analysis was beyond the scope of the present work.

4.3 The test website indicates that the LSA and one other band is required for a positive result. This study uses 2 bands in addition to LSA. Why the difference? Requiring 2 bands for this study would likely lead to better specificity determinations.

The use other antigen bands in addition to LSA was specified in our manuscript and scoring is better clarified in the revision in L152-158 and L170-175. The cause of the reference to

‘one other band’ is not on the website. It seems likely a misunderstanding of the statement that ≥1 antigen band from of any of the nine homologous antigen bands used for P23/OspC (for example) is considered to indicate the presence of antibodies to P23 in the test serum.

4.4 LD antibody testing results 4.4 More detail is needed on the serum panels used. Were any of the patient vs control classifications based on LD antibody testing results?

All sera (control and patient) from the CDC and BALDB were tested in a blinded manner by us. Details of how the control sera from LD endemic and non-endemic sera were classified are provided in the cited references. The sera from persons with non-LD infections and other potentially confounding medical conditions would not have been tested for LD by STTTs. Exception to this are the IGeneX sera from other tick-borne diseases (as stated in Table 1) that were used as controls for specificity. These would have been tested for LD by STTTs at the time they were received and would have negative for LD, i.e. for coinfection. However, they were not previously tested by the IBs used here.

4.5 The publication by Porwancher, et al. (Pathogens. 2024:13) suggests that specificity may not be as good as the 2-tiered protocol with CDC interpretive criteria. This should be addressed in the discussion.

The interesting study by Porwancher et al 2024 Pathogens. 2023 Oct 26;12(11):1282. doi: 10.3390/pathogens12111282.PMID: 38003747, had serious drawbacks that were detailed in our published response article– Shah et al. Pathogens 2024, 13, 352. https://doi.org/10.3390/pathogens13050352, PMID: 38787204 and which was responded to in Porwancher et al. Pathogens. 2024 13:353. doi: 10.3390/pathogens13050353. PMID: 38787205. We respectfully suggest that it not helpful to revisit this particular issue here. The present data show that the specificities of IB tests used here are indeed comparable to the STTTs.

Reviewer #4 (Minor Comments for Author (Required)):

4.6 The authors claim reduced labor with the line blot tests. However, there are MTTT assays on automated immunoassay platforms that have minimal hands-on time that in lieu of line blot automation would likely have less labor involved. In addition, without automation how can the line blot format be incorporated into a high-volume testing environment?

This comment is appreciated. We have modified the claim regarding labour, time and costs and refer to simplification of testing in line 400-405. Line immunoblots can be readily machine read for high volume testing and we have worked on this. Automated reading was

not applied to the data used in the manuscript. Our testing volumes do not justify routine automated reading at present.

4.7 How was Protein L and its corresponding band intensity validated as a suitable control for interpreting band presence?

This was done with positive and negative LD sera from the CDC's LD research panel to determine the optimal concentration of protein L that gave the best specificity and sensitivity. This now explained better in the revised manuscript L125-130.

4.8 Lines 146 - 149: Do the assay instructions for use provide for the use of alternate interpretive approaches to call positive v negative results?

When the iDart IB tests are marketed with inclusion of scoring antigens based on the CDC STTT criteria, appropriate assay instructions will be provided.

4.9 Line 223: A serum was considered true positive if a subsequent sample was also positive. How many samples did this apply to. It seems that a patient with a false positive result may continue to display this result regardless of when it was collected. Please justify this approach for discrepancy resolution.

In the revision we have deleted reference to the use of 156 convalescent patient sera Bay Area Lyme Foundation Biobank (Table 1 and throughout) to avoid confusion with the acute phase sera. Convalescent phase sera results were not essential for Discrepancy analysis as 17 of 21 sera classed as True Positives had EM lesions and the other 4 were positive by the two EIA criteria (L348-355).

4.10 Lines 250 - 255: More clarity is needed in how these experiments were run. Does this mean that the "blocked" conjugates did not result in a positive result??

Thank you for this comment. The answer to the question is yes and we have explained this simple experiment better in the revised manuscript in L180-188 Methods and L257-262 under Results.

4.11 Lines 301 - 304: The lack of statistically significant improvement in sensitivity should be addressed in the discussion.

This related to findings with the CDC serum panel only. We appreciate this comment and have better explained this in the revision in L305-322 more appropriately under Results.

4.12 Lines 361 - 363: This sentence is in contrast to the overall findings that the line blot test improved sensitivity was limited to stage 1 disease as indicated in lines 364 - 365.

We appreciate this comment and have explained it better in the revision in L380-388.

Re: Spectrum00199-26R1 (Single Step Immunoblot Tests with Recombinant Protein Antigens for Detecting IgG and IgM Antibodies in Lyme Disease)

Dear Dr. Ranjan Ramasamy:

Your manuscript has been accepted, and I am forwarding it to the ASM production staff for publication. Your paper will first be checked to make sure all elements meet the technical requirements. ASM staff will contact you if anything needs to be revised before copyediting and production can begin. Otherwise, you will be notified when your proofs are ready to be viewed.

Data Availability: ASM policy requires that data be available to the public upon online posting of the article, so please verify all links to sequence records, if present, and make sure that each number retrieves the full record of the data. If a new accession number is not linked or a link is broken, provide production staff with the correct URL for the record. If the accession numbers for new data are not publicly accessible before the expected online posting of the article, publication may be delayed; please contact ASM production staff immediately with the expected release date.

Publication Fees: For information on publication fees and which article types have charges, please visit our website. We have partnered with Copyright Clearance Center (CCC) to collect author charges. If fees apply to your paper, you will receive a message from no-reply@copyright.com with further instructions. For questions related to paying charges through RightsLink, please contact CCC at ASM_Support@copyright.com or toll free at +1-877-622-5543. CCC makes every attempt to respond to all emails within 24 hours.

ASM Membership: Corresponding authors may join or renew ASM membership to obtain discounts on publication fees. Need to upgrade your membership level? Please contact Customer Service at Service@asmusa.org.

Embargo Policy: A press release may be issued as soon as the manuscript is posted on the Spectrum Latest Articles webpage. The corresponding author will receive an email with the subject line "ASM Journals Author Services Notification" when the article is available online.

The ASM Journals program strives for constant improvement in our submission and publication process. Please tell us how we can improve your experience by taking this quick Author Survey.

Thank you for submitting your paper to Spectrum.

Sincerely,
Travis Bourret
Editor
Microbiology Spectrum
